# Synthesizing non-natural parts from natural genomic template

**DOI:** 10.1186/1754-1611-3-2

**Published:** 2009-02-03

**Authors:** Pawan K Dhar, Chaw Su Thwin, Kyaw Tun, Yuko Tsumoto, Sebastian Maurer-Stroh, Frank Eisenhaber, Uttam Surana

**Affiliations:** 1 Synthetic Biology Lab, RIKEN Advanced Sciences Institute, Yokohama, 230-0045, Japan; 2 Biomolecular Function Discovery Division, Bioinformatics Institute, Agency for Science, Technology and Research (A*STAR), 30 Biopolis Street, 138673, Singapore; 3 Cell Cycle control Lab, Institute of Molecular and Cellular Biology, 61 Biopolis Drive, 138673, Singapore

## Abstract

**Background:**

The current knowledge of genes and proteins comes from 'naturally designed' coding and non-coding regions. It would be interesting to move beyond natural boundaries and make user-defined parts. To explore this possibility we made six non-natural proteins in *E. coli*. We also studied their potential tertiary structure and phenotypic outcomes.

**Results:**

The chosen intergenic sequences were amplified and expressed using pBAD 202/D-TOPO vector. All six proteins showed significantly low similarity to the known proteins in the NCBI protein database. The protein expression was confirmed through Western blot. The endogenous expression of one of the proteins resulted in the cell growth inhibition. The growth inhibition was completely rescued by culturing cells in the inducer-free medium. Computational structure prediction suggests globular tertiary structure for two of the six non-natural proteins synthesized.

**Conclusion:**

To our best knowledge, this is the first study that demonstrates artificial synthesis of non-natural proteins from existing genomic template, their potential tertiary structure and phenotypic outcome. The work presented in this paper opens up a new avenue of investigating fundamental biology. Our approach can also be used to synthesize large numbers of non-natural RNA and protein parts for useful applications.

## Background

Organisms use Nature's inventory of materials and designs for living. The raw material mostly comes in the form of DNA, RNA and protein. DNA, a repository for long-term storage of genetic instructions, comprises of genes and intergenic regions. While genic regions have been thoroughly investigated in the past, intergenic regions have received increased attention recently [1-4]. It would be interesting to mine intergenic regions for unidentified genes and also use them for making novel proteins.

Here we present a simple and scalable approach of making non-natural proteins from the 'not-coding' intergenic regions. The term 'not-coding' has been used in the context of not-naturally-designed for making proteins. As against the previously described approaches of chemically synthesizing randomized protein sequences [5,6] adding tolerated point mutations to natural proteins [7], generating polypeptide sequences by combinatorial shuffling [8], improving protein functions through directed evolution [9] we used the existing genomic template of *E. coli *to make non-natural proteins.

**Figure 1:**
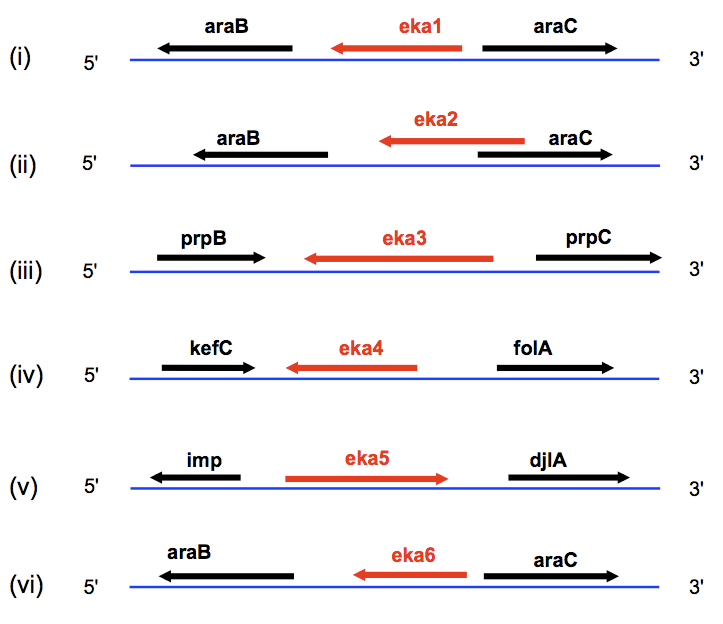
**eka neighbourhood**. The nearest neighbourhood scan of the eka sequences. All the sequences are in the intergenic region with the exception of eka2 that shows 32% sequence overlap with araC gene.

As a first step, six unique intergenic regions (> 100 bases in length), with no history of transcription, were randomly selected (Table 1). Of the six samples, five came from non-overlapping intergenic regions (Fig 1). One more sequence overlapping with a coding region was deliberately added to explore the general applicability of our method. Following criteria were adopted for selecting genome sequences: (a) the not-coding feature of sequences based on the absence of complete similarity with known proteins (b) sequences of different sizes and orientations. All the sequences were cloned using pBAD vector and expressed after transfection into E. coli. Expression was confirmed using western blot. The non-natural proteins were called *eka*, meaning 'first' in sanskrit. Cell growth and shape were used as convenient phenotypic indicators to study the effect of their intracellular expression. Standard computational methods were used to predict potential structures of the proteins synthesized.

**Table 1:** Description of eka sequences

ID	Length *a*	Start – End	Overlap	Vector sequence		Total *a+b+c*	% vector contribution	Protein		e-value	Bit score	GC ratio	
				*b*	*c*			Aa	M.W.			(i)	(ii)
eka1	104	70,283 – 70,386	No	381	157	642	83.8	214	23.5	>10	*	39.4	50.0

eka2	138	3,651,282 – 3,651,704	Yes, 32%	381	90	609	77.3	203	22.1	> 10	*	42.0	48.3

eka3	432	348,779 – 349,210	No	381	90	903	52.1	301	33.7	6 e-04	46.2	47.0	48.6

eka4	105	49,681 – 49,785	No	381	90	576	81.7	192	20.9	>10	*	49.5	50.0

eka5	141	57,173 – 57,313	No	381	90	612	76.9	204	22.2	> 10	*	43.2	50.8

eka6	96	70,285 – 70,380	No	381	90	567	83.1	189	20.5	>10	*	39.6	48.3

Start-end indicates genomic location of the selected sequences. 'a' indicates the length of the original genomic insert, 'b' and 'c' indicate vector contributed prefix and suffix DNA sequences respectively. Total (a+b+c) indicates the entire DNA sequence expressed into proteins. The pBAD vector contribution to the final protein sequence is indicated in percentages. Aa indicates the number of amino acid residues of the synthesized protein. M.W. refers to the Isotopically Averaged Molecular Weight calculated in kiloDaltons (kDa). (i) indicates GC ratio of the genomic insert, and (ii) indicates GC ratio of the complete DNA sequence (vector + genomic DNA) expressed into proteins. The large e-value and extremely small bit score approaching zero (*) indicates very low sequence similarity of eka proteins to the known protein sequences.

## Results and discussion

**Figure 2:**
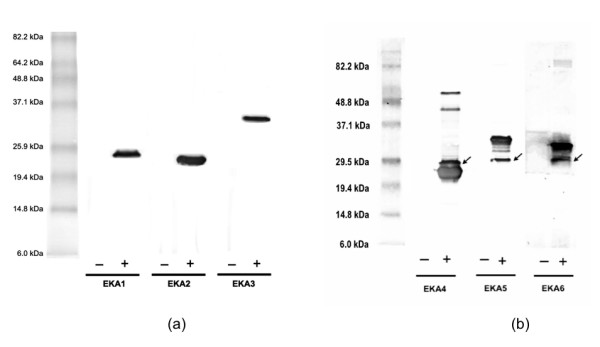
**western blot**. The western blot of (a) EKA 1-EKA 3, (b) EKA 4 – EKA 6 proteins. See ref. [26] and the method section for details. The – sign indicates the negative control and + sign indicates induced expression of EKA protein.

**Figure 3:**
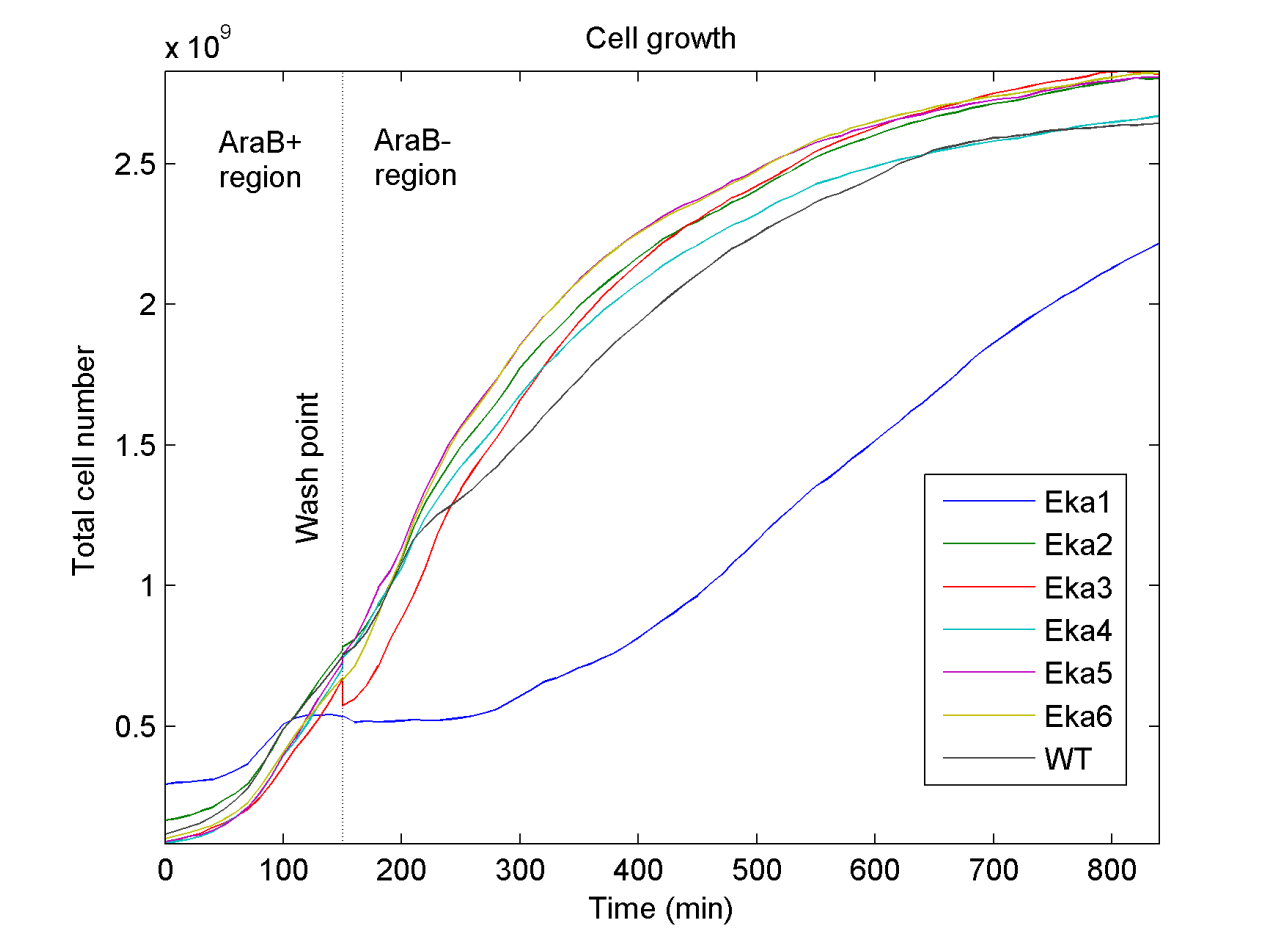
**cell growth**. The growth-plot of the wild type (WT) and transformed *E. coli *cells (Eka1-Eka6). A tiny nick at the AraB+/AraB- boundary indicates loss of eka3 transformed cells at the wash step.

The natural non-protein-coding property of eka sequences was confirmed by sequence similarity comparisons using BLASTP against the non-redundant protein database of NCBI. The entire full-length EKA sequences did not fully resemble any known naturally occurring proteins (Table 1). Amplified sequences and enzyme digests of the recombinant pBAD vector matched expected molecular weights. The presence and correct orientation of eka sequences (in the pBAD vector) was confirmed by sequencing and gel electrophoresis. The overall length of the protein sequences was found to be longer than the expected. This was due to contribution from the pBAD vector to the final protein sequences (Table 1). The Western blot (Fig 2) confirmed expression of EKA 1–6 proteins. Of six proteins expressed intracellularly, EKA1 showed significant growth inhibitory effects whereas EKA2 – EKA6 expression did not impact the cell growth (Fig 3). We do not yet know the effect of prefix and suffix sequences on the physiological behavior or folding pattern of the final protein sequences. It is interesting to note a shift in the GC content from 39.4 – 49.5 (original genomic insert) to 48.3 – 50.8 (after insertion into the vector). It is not known if the shift in GC content is one of the reasons for eka protein expression in E. coli with an average GC content of 50.8% [10]. A future step will be producing proteins without prefix and suffix sequences and compare folding pattern and phenotypes.

**Figure 4:**
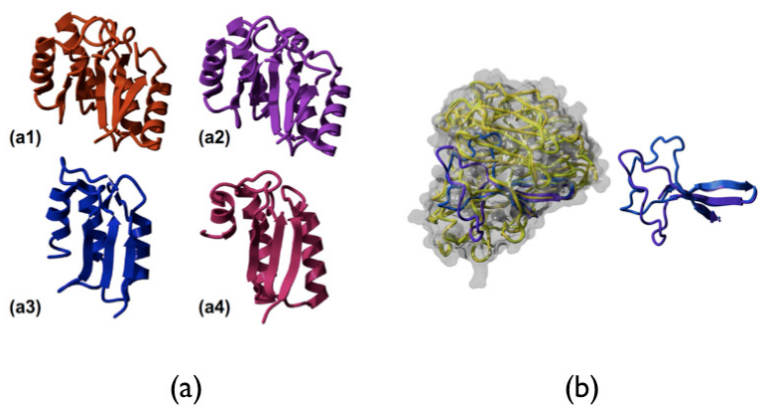
**tructural models of proteins**. (a) Structural models of the EKA3 protein based on the PDB templates in order of their ranking by 3D-Jury: (a1) 1ub3 (a2) 1mzh (a3) 2dxn and (a4) 2hy1. (b) Structural models of the EKA5 protein based on the PDB templates 2htv (lila) and 2ht5 (blue). Left side: The two structural models in the context of the full structure of their templates 2htv (yellow) and 2ht5 (green).

Of six sequences only two i.e., EKA3 and EKA5, produced results that could be interpreted as being partially compatible with forming a tertiary structure. In the resulting four models of EKA3: 1ub3 [11], 1mzh [Tan AY, Smith PC; Crystal Structure of Aquifex Aeolicus Aldolase, Unpublished] 2dxn [12], 2hy1 [13]. (Fig 4a), we observed a recurring consensus pattern of alternating helix and beta strand that assembles into a larger structure with a combined beta sheet on one side and the packed helices on the other. The difference in the width of the beta strands and helices imposes a curvature on the structure leaving the beta sheet in the concave inside and the helices in the convex outside. While in the representative template Aldolase (PDB:1mzh) this pattern leads to full closure of the beta sheet into a beta barrel, EKA3 has less repeating units and, hence, covers approximately half of the full template structure. Though the helical outside surface of our models consists of mainly hydrophilic residues pointing into the solvent, there are some hydrophobic residues in the beta sheets pointing to the inside of the half beta barrel, which may be partially exposed to solvent and hence could cause problems for folding of this structure.

In EKA5 (Fig 4b), weak similarity to the beta propeller fold of viral neuroaminidases was suggested by using the PDB-Basic tool from the 3D-Jury authors [14]. When modelling EKA5 onto the 2 predicted templates (PDB: 2htv and 2ht5, [15] using Modeller [16], it becomes apparent that the aligned portion of EKA5 only covers 3 out of 4 beta strands that would normally form a beta sheet representing one of six blades of the overall propeller structure (Fig 4b). Correct folding depends on proper stacking of the blades including hydrophobic contacts that would indicate that a single blade alone, as predicted for EKA5, would not form a stable structure. An interesting question is whether EKA5 single blades could eventually homo-polymerize into a full propeller, when over-expressed. However, such speculations can only be answered through further experimental structural studies.

Most of the currently known protein functions require folding of a protein sequence into a globular structure. Hence, we wanted to investigate if the sequences could principally adopt a known fold. While pure *ab initio *structure prediction is still in its infancy, the most successful current methods strongly rely on sequence similarity for fold recognition. However, we are dealing with new unknown sequences not expected to have clear homologues. Our method of choice was to try several possibilities and take a consensus of the predictions [17]. Notably, threading methods gave more consistent results (more template hits were similar to each other), which makes sense since threading methods emphasize more on compliance with the biophysical needs of a sequence fitting into the structure rather than depending on similarity to sequences of known structures. Only for the longest of the 6 sequence inserts, EKA3 (143 amino acids), a globular tertiary structure was predicted. EKA5 (47 amino acids) also showed similarity to a known fold, however, only to one of its substructures not known to form a stable structure on its own. Similarly, the other four, EKA1 (33 amino acids), EKA2 (46 amino acids), EKA4 (35 amino acids) and EKA6 (32 amino acids) appear too short to form complex tertiary structures on their own. At best we find similarities to not more than a single helix. Furthermore, low complexity predictions [18] over large parts of their sequence are an additional indicator of absence of globular structure. On the other hand, the proposed structure for EKA3 is consistent among the models derived from the 4 top-ranked hits, adding support to the prediction, and the inter-model variability (Fig 4a) allows estimating the maximal accuracy that can be expected in this case. However, structure predictions in the absence of sequence similarity remain notoriously difficult at this time and experimental validation is needed to confirm validity of our models.

We do not disregard the possibility that some of the EKA genes may turn out to be real in some organisms or may represent evolutionary remnants of what-was-once a functional sequence. In fact, in one of the previous studies, expression for 4052 coding transcripts and 1102 additional transcripts in the intergenic regions of the E. coli genome was identified using the whole genome array [19]. However, intentional conversion of these sequences to synthesize non-natural proteins is a novel attempt, to our best knowledge. One could ask why Nature didn't sample these genomic regions? And if Nature indeed sampled these regions – were these proteins discarded? If yes, why? To answer such questions one must synthesize more non-natural proteins and study their impact on cell physiology. It would be interesting to sample conserved intergenic regions, subsets of introns, overlapping regions, and so on and study the impact of making novel RNA and protein parts. Given that 98.5% of human genome is made of intergenic regions, it would be useful to mine this enormous resource to make non-natural parts for useful applications. It has not escaped our attention that our approach can be extended to make non-natural RNA parts, both coding and non-coding.

Interestingly, several studies point to the evolution-driven conversion of not-coding regions to coding regions [20-24]. However, our work demonstrates a user-defined conversion leading to the synthesis of non-natural parts. It would be relevant to ask: how to evaluate functions of genes 'not naturally needed for survival'. The traditional approaches of gene knockout and down-regulation of expression are unattractive since organisms don't need these parts by default. In our opinion, expressing such sequences under the control of a strong promoter, followed by microarray analysis could help identify interactions and pathways through which such non-natural parts act.

Furthermore, non-natural proteins that are stably expressed can be systematically tested if they adopt new folds or functions of any kinds. Besides looking at these non-natural proteins in isolation, different effects could possibly be obtained by combining them with known domains. In theory, it could be possible to derive novel synthetic multi-domain proteins in a combinatorial fashion. Given that our analyzed examples indicate both non-coding intergenic and coding but out-of-frame segments as suitable candidates for producing variants of new proteins, the imaginable number of potential new RNA and protein parts and their combinations is enormous.

It is worth noting that our work does not describe an approach for rationally designing RNA and proteins parts based on higher-level parameters. Our approach resembles a semi-random strategy of synthesizing non-natural parts, followed by functional analysis. Although we were successful in expressing all the six sequences, we do not know the boundary conditions of this approach, if any. To answer this question, one should make proteins from genome regions of different lengths, origins and features.

## Conclusion

To the best of our knowledge this is the first report that describes artificial synthesis of protein parts from genomic regions not naturally utilized to make proteins. It would be interesting to extend this study to synthesize and characterize non-natural RNA parts. The cell-free synthesis of non-natural parts can be used in situations where their intracellular synthesis results in cell death. The other important issue, addressed through this work, is the prediction of potential tertiary structures of non-natural proteins. Though initial computational analysis indicates several potential structures, experimental study is needed to confirm these predictions. In future, an extensive study is required to uncover existence of novel structures 'possibly embedded' in the genome. Finally, our approach can be used to make novel enzymes, transcription factors, receptor proteins and so on.

## Methods

**Figure 5:**
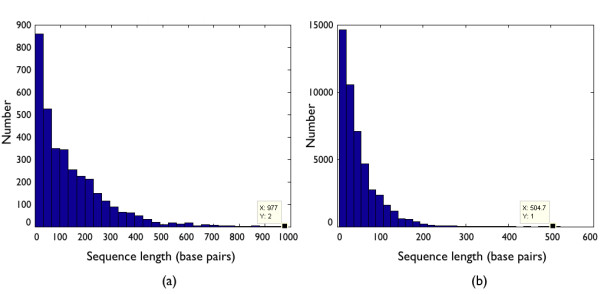
**E. coli intergenic regions**. Distribution of the contiguous intergenic regions in E. coli (a) that include stop codons and (b) without stop codons. Note an increase in the longer genomic fragments in the sample (a) available for making non-natural proteins.

**Figure 6:**
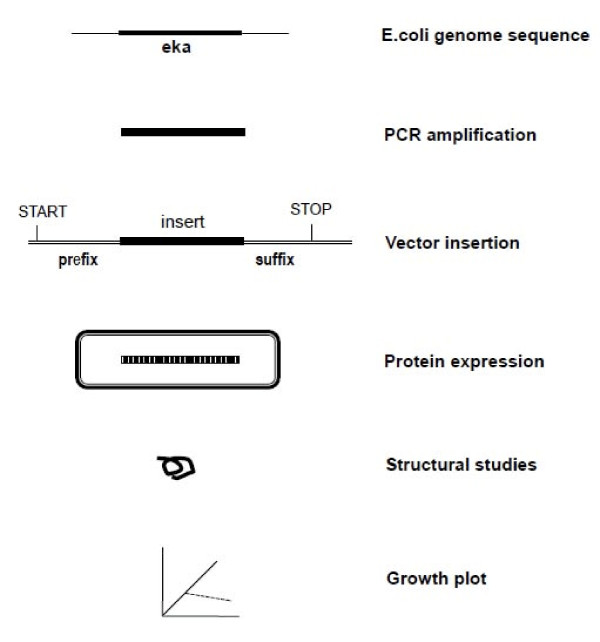
**Overview of the method**. General scheme of producing proteins from not-naturally-coding DNA sequences.

**Figure 7:**
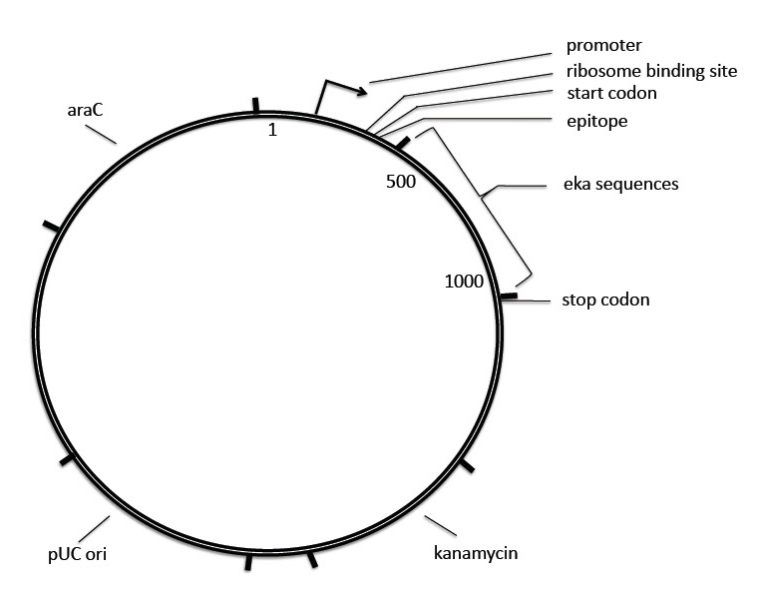
**vector construction**. Schematic diagram of the Vector showing the site of insertion, Ribosome Binding site, start codon, epitope and stop codon.

Six eka sequences were chosen on the basis of the absence of a complete set of promoter, start and stop signals. In addition to sampling intergenic regions, overlapping regions of coding and intergenic sequences were also considered to broaden the scope of the work. From a large number of possibilities that exist (Fig 5) contiguous sequences were randomly chosen at an arbitrary cut-off value of ~100 bases. All the eka sequences (Additional file 1) were computationally translated into amino acid sequences. These sequences were BLASTed against the NCBI NR protein sequence database to find similarity to known proteins, if any. PSI-BLAST [25] was used at the e-value cut off 10. The *E. coli *K12 (MG1655) strain, provided by the National Institute of Genetics (NIG, Japan), were grown in the LB growth medium at 37°C. The transformed cells were cultured in kanamycin-supplemented LB medium (50 μg·ml-1). Genomic DNA was purified by Wizard^® ^Genomic DNA Purification Kit. PCR amplification was performed with the forward and reverse primers by using the *E. coli *genome as a template. The PCR products of the sequences were confirmed by gel electrophoresis. The overall approach (Fig. 6) essentially comprised selecting the desired sequence and amplifying and inserting the sequences into a pBAD202/D-TOPO vector (Invitrogen). The pBAD vector provided a ready-to-use template, an inducible promoter, start and stop codons, for expression of sequences (Fig 7). Inserting eka sequences downstream of the "promoter and start codon" and upstream of the stop codon generated coding sequences. The directional insertion of eka sequences in the pBAD topo vector was achieved by following the company's protocol. The presence and orientation of inserts was confirmed by sequencing and gel electrophoresis. The recombinant pBAD vector was used for the transformation of the One Shot^® ^TOP10 chemically competent MG1655 E. coli cells by using Invitrogen's standard protocol. Colonies were screened on kanamycin (50 μg·ml-1)-supplemented LB medium. Protein expression in the transformed MG1655 cells was induced by adding 0.02% arabinose to the culture medium. The EKA proteins in the transformed cells were detected by western blotting (WesternBreeze kit, Invitrogen). The expression of proteins was visualized by western blot according to the standard protocols [26]. For each sample, 10–20 μl of the proteins were electrophorsed using 12% SDS PAGE for 70 min at 200 V. Proteins were transferred from gel to polyvinyldifluoride (PVDF) membrane for 1 h at 100 V. Western blot was performed using WesternBreeze^® ^chromogenic western blot immunodetection kit according to the manufacturer's protocol. After 1 h blockade of nonspecific binding sites by blockers, the PVDF membrane was incubated for 15 h at 4°C with a mouse anti-cleaved Anti-ThioTM antibody (1:20,000) followed by 1 h incubation with an alkaline phosphatase-conjugated anti-mouse IgG secondary antibody. Immunoblots were developed using chromogenic substrate for 10 min and the membrane was air-dried overnight. The chromogenic substrate was a mixture of BCIP (5-Bromo-4-Chloro-3-Indolyl phosphate) and NBT (Nitroblue Tetrazolium Salt). The solution provided by Invitrogen was diluted 8 times with distilled water and then used.

The positive (pBAD/D/lacZ, Invitrogen) and negative controls (i.e. without eka sequences) were used to validate the expressions of EKA proteins. The pBAD202/D/lacZ vector was used as a positive control, and the pBAD202/D-TOPO vector without eka sequence was used as a negative control. Cell growth was automatically monitored every 10 minutes for 10 hours using an automated multiplate reader (Tecan Plate Reader, Magellan 200) at 37°C. The growth inhibitory effect of EKA1 was rescued by removing the inducer i.e., washing and re-culturing cells in arabinose (-) medium (Fig 3).

To investigate the possibility of EKA proteins folding into globular structures, all the six protein sequences were submitted to the consensus structure prediction method, 3D-Jury [14]. The algorithm identifies consensus structural units shared among templates suggested by a wide range of established structure prediction servers. In the case of EKA3, all 4 top-ranked hits came from predictions by the threading method mGenThreader [27]. Since these hits are structurally related, we chose each of them as separate templates for modeling using the Software Modeller [16] (version 9.1) to gauge the structural variability of similar predictions. Figures of the structures were generated using Yasara application [28].

## Competing interests

The authors declare that they have no competing interests.

## Authors' contributions

PKD conceived the original concept, designed experiments, analyzed the data and wrote the paper. CST and KT carried out experiments, SMS and FE performed computational structure analysis of proteins and wrote the protein structure part of the paper, US advised during experiments and analyzed data. All authors reviewed and gave final approval of this version of the paper.

## Supplementary Material

Additional file 1 **eka1 – 6 SEQ**. The protein sequence of eka1-6 genes. Red color indicates the computed amino-acid sequence of the original genomic insert. Click here for file
