# Supplementary material for: Synthesizing non-natural parts from natural genomic template
Source: J Biol Eng. 2009 Feb 3;3:2. doi: 10.1186/1754-1611-3-2 (PMC2642765; doi:10.1186/1754-1611-3-2)
Supplement: Additional file 1 — eka1 – 6 SEQ. The protein sequence of eka1-6 genes. Red color indicates the computed amino-acid sequence of the original genomic insert. [file 1754-1611-3-2-S1.pdf]

EKA protein sequences – red color indicates genomic insert

EKA1 protein sequence (genomic insert + vector sequence)

MGSDKIIHLTDDSFDTDVLKADGAILVDFWAHWCGPCKMIAPILDEIADEYQGKLTV
AKLNIDHNPGTAPKYGIRGIPTLLLFKNGEVAATKVGALSKGQLKEFLDANLAGSGS
GDDDDKLGIDPFTTFHTPTIQRRNQLSILHQTLPSLRLLLALLANPTGRASSSLKVS
LSLTLSSVSILRVPVIITITIEFKRSPAWLFWRMREDFQPDTD

EKA2 protein sequence (genomic insert + vector sequence)

MGSDKIIHLTDDSFDTDVLKADGAILVDFWAHWCGPCKMIAPILDEIADEYQGKLTV
AKLNIDHNPGTAPKYGIRGIPTLLLFKNGEVAATKVGALSKGQLKEFLDANLAGSGS
GDDDDKLGIDPFTKRVSRQQGIILRFSHTFHTPTIQRRNQLSILHQTLPSLRLLLAL
LAKGELKLEGKPIPNPLLGLDSTRTGHHHHHH

EKA3 protein sequence (genomic insert + vector sequence)

MGSDKIIHLTDDSFDTDVLKADGAILVDFWAHWCGPCKMIAPILDEIADEYQGKLTV
AKLNIDHNPGTAPKYGIRGIPTLLLFKNGEVAATKVGALSKGQLKEFLDANLAGSGS
GDDDDKLGIDPFTVGSRLLSDATLCVLSDLQFSSPRRPDKSRQRRIRHRLSDATLCV
LSDLQFSAPRRPDKSRQRRIRHRLSDATLCVLSDLQFSAPRRPDKSRQRRIRHRLSD
ATPSVLSDLQFSAPRRPDKSRQRRIRHRLSNTINGRFLFHLATKGELKLEGKPIPNP
LLGLDSTRTGHHHHHH

EKA4 protein sequence (genomic insert + vector sequence)

MGSDKIIHLTDDSFDTDVLKADGAILVDFWAHWCGPCKMIAPILDEIADEYQGKLTV
AKLNIDHNPGTAPKYGIRGIPTLLLFKNGEVAATKVGALSKGQLKEFLDANLAGSGS
GDDDDKLGIDPFTVKRKPSSTGARMMLRKILYSAGESTLSPWTRRITMKGELKLEGK
PIPNPLLGLDSTRTGHHHHHH

EKA5 protein sequence (genomic insert + vector sequence)

MGSDKIIHLTDDSFDTDVLKADGAILVDFWAHWCGPCKMIAPILDEIADEYQGKLTV
AKLNIDHNPGTAPKYGIRGIPTLLLFKNGEVAATKVGALSKGQLKEFLDANLAGSGS
GDDDDKLGIDPFTTRHLYASVNPVRIASVVLGTALNDRYDNANYRRCPTIDRSRKTV
KAPKGELKLEGKPIPNPLLGLDSTRTGHHHHHH

EKA6 protein sequence (genomic insert + vector sequence)

MGSDKIIHLTDDSFDTDVLKADGAILVDFWAHWCGPCKMIAPILDEIADEYQGKLTV
AKLNIDHNPGTAPKYGIRGIPTLLLFKNGEVAATKVGALSKGQLKEFLDANLAGSGS
GDDDDKLGIDPFTHTPTIQRRNQLSILHQTLPSLRLLLALLANPTKGELKLEGKPIP
NPLLGLDSTRTGHHHHHH
